# Supplementary material for: Zika Virus Immunoglobulin G Seroprevalence among Young Adults Living with HIV or without HIV in Thailand from 1997 to 2017
Source: Viruses. 2022 Feb 10;14(2):368. doi: 10.3390/v14020368 (PMC8878279; doi:10.3390/v14020368)
Supplement: Supplementary file 1 [file viruses-14-00368-s001.zip › viruses-1556650-SI.pdf]

Supplementary Table S1. Characteristics of the study population.

**Characteristics**		**Overall (n=1648)**		**HIV-infected pregnant women (1997–2000) (n=386)**		**All pregnant women (1997–2017) (n=895)**		**Subjects presenting for HIV testing (2015–2017) (n=353)**[1]	
		**n/N or n**	Percentage or median (IQR)	**n/N or n**	Percentage or median (IQR)	**n/N or n**	Percentage or median (IQR)	**n/N or n**	Percentage or median (IQR)
Sex	Female	1,464/1,648	88.8	386/386	100.0	895/895	100.0	169/353	47.9
	Male	174/1,648	10.6	0/386	0.0	0/895	0.0	174/353	49.3
	Other	10/1,648	0.6	0/386	0.0	0/895	0.0	10/353	2.8
Age (years old)		1,648	22.0 (20.0, 23.0)	386	22.0 (20.1, 23.0)	895	22.0 (20.0, 23.0)	353	22.0 (20.0, 23.0)
Pregnancy (denominator: females)		1,295/1,464	88.5	386/386	100.0	895/895	100.0	-[2]	-[2]
Gestational age (weeks)		1,262	25.0 (16.7, 29.7)	386	21.4 (16.9, 25.3)	895	27.3 (21.3, 31.3)	-[2]	-[2]
Region of birth	Central	250/1,522	16.4	46/339	13.6	171/823	20.8	60/345	17.4
	Northern	757/1,522	49.7	156/339	46.0	254/823	30.9	252/345	73.0
	Northeastern	173/1,522	11.4	5/339	1.5	158/823	19.2	11/345	3.2
	Eastern	273/1,522	17.9	125/339	36.9	192/823	23.3	3/345	0.9
	Western	25/1,522	1.6	0/339	0.0	15/823	1.8	10/345	2.9
	Southern	38/1,522	2.5	7/339	2.1	27/823	3.3	9/345	2.6
	Foreign country	6/1,522	0.4	0/339	0.0	6/823	0.7	0/345	0.0
Region of enrollment	Central	250/1,644	15.2	58/386	15.0	219/895	24.5	12/351	3.4
	Northern	868/1,644	52.8	168/386	43.5	277/895	31.0	335/351	95.4
	Northeastern	70/1,644	4.3	0/386	0.0	68/895	7.6	2/351	0.6
	Eastern	413/1,644	25.1	152/386	39.4	292/895	32.6	0/351	0.0
	Western	7/1,644	0.4	0/386	0.0	6/895	0.7	1/351	0.3
	Southern	36/1,644	2.2	8/386	2.1	33/895	3.7	1/351	0.3
Education	Higher than bachelor's degree	5/1,638	0.3	0/383	0.0	0/892	0.0	5/350	1.4
	College/ University	369/1,638	22.5	25/383	6.5	90/892	10.1	258/350	73.7
	High school	188/1,638	11.5	24/383	6.3	87/892	9.8	64/350	18.3
	Secondary school/ Vocational certificate	394/1,638	24.5	95/383	24.8	283/892	31.7	19/350	5.4
	Primary school	482/1,638	29.4	164/383	42.8	302/892	33.9	1/350	0.3
	Lower than primary school	175/1,638	10.7	75/383	19.6	119/892	13.3	1/350	0.3
	Others	25/1,638	1.5	0/383	0.0	11/892	1.2	2/350	0.6
Marital status	Living with partner	834/892	93.5	372/384	96.9	834/892	93.5	n.a.[3]	n.a.[3]
	Divorced/ Not living with partner/ Widowed/ Single	53/892	5.9	12/384	3.1	53/892	5.9	n.a.[3]	n.a.[3]
	Others	5/892	0.6	0/384	0.0	5/892	0.6	n.a.[3]	n.a.[3]
Number of household members	1 (Living alone)	101/611	16.5	n.a.[3]	n.a.[3]	3/260	1.2	98/351	27.9
	2 people	136/611	22.3	n.a.[3]	n.a.[3]	77/260	29.6	59/351	16.8
	3 people	96/611	15.7	n.a.[3]	n.a.[3]	40/260	15.4	56/351	16.0
	4 people	110/611	18.0	n.a.[3]	n.a.[3]	54/260	20.8	56/351	16.0
	More than 4 people	168/611	27.5	n.a.[3]	n.a.[3]	86/260	33.1	82/351	23.4
Multiple partner		77/236	32.6	n.a.[3]	n.a.[3]	n.a.[3]	n.a.[3]	77/236	32.6
Occupation	Unemployed or Housewife	487/1,603	30.4	25/386	6.5	388/856	45.3	10/350	2.9

	Agriculturist/ Fishery	176/1,603	11.0	63/386	16.3	70/856	8.2	0/350	0.0
	Commercial/ Private business/ Self-employed	128/1,603	8.0	42/386	10.9	86/856	10.1	8/350	2.3
	Office man	152/1,603	9.5	124/386	32.1	125/856	14.6	0/350	0.0
	Labor/ Housekeeper	292/1,603	18.2	121/386	31.3	161/856	18.8	0/350	0.0
	Student	303/1,603	18.9	2/386	0.5	8/856	0.9	292/350	83.4
	Others	65/1,603	4.1	9/386	2.3	18/856	2.1	40/350	11.4
Risk behavior	Alcohol consumption	290/404	71.8	24/24	100.0	56/56	100.0	234/348	67.2
	Smoking	59/349	16.9	0/24	0.0	0/56	0.0	59/349	16.9
	Drug use	73/351	20.8	0/24	0.0	0/56	0.0	73/351	20.8
	Any of these	301/407	74.0	24/24	100.0	56/56	100.0	245/351	69.8
Infection status	Anti-HIV positive	844/1,645	51.2	386/386	100.0	840/892	94.2	4/353	1.1
	HIV RNA load (log_{10} copies/mL)	838	3.88 (3.21, 4.46)	386	3.92 (3.32, 4.40)	834	3.87 (3.21,4.45)	4	4.95 (4.39, 5.70)
	HIV RNA load among pregnant women (log_{10} copies/mL)	834	3.87 (3.21, 4.45)	-[2]	-[2]	-[2]	-[2]	-[2]	-[2]
	HBsAg positive	111/1,245	8.9	28/385	7.3	103/892	11.6	8/353	2.3
	Anti-HCV positive	22/1,246	1.8	13/384	3.4	21/893	2.4	1/353	0.3
	Syphilis positive	3/353	0.8	n.a.[3]	n.a.[3]	n.a.[3]	n.a.[3]	3/353	0.9
Blood chemistry testing	Fasting blood sugar (mg/dL)	112	82 (73, 91)	17	90 (85, 109)	112	82 (73, 91)	n.a.[3]	n.a.[3]
	Cholesterol (mg/dL)	248	217 (180, 258.5)	32	197 (160.5, 225)	284	217 (180, 258.5)	n.a.[3]	n.a.[3]
	AST (IU/L)	184	21.0 (17, 29.5)	32	30.5 (21.5, 46)	184	21 (17, 29.5)	n.a.[3]	n.a.[3]
	ALT (IU/L)	866	14.0 (10.0, 20.0)	385	14 (10, 20)	883	14 (10, 20)	n.a.[3]	n.a.[3]
Hematological testing	Hemoglobin (g/dL)	893	10.8 (11.6, 10)	384	10.6 (9.9, 11.4)	893	10.8 (10, 11.6)	n.a.[3]	n.a.[3]
	Hematocrit (%)	895	33.0 (35.0, 30.9)	386	33.0 (30.9, 35.1)	895	33 (30.9, 35)	n.a.[3]	n.a.[3]
	RBC count (million cells/mL)	516	4.0 (3.6, 4.4)	129	3.97 (3.54, 4.40)	516	3.99 (3.61, 4.41)	n.a.[3]	n.a.[3]
	Platelet count (thousand/mm^3)	596	78.5 (180.0, 258.5)	87	241 (197, 286)	596	263 (221, 309)	n.a.[3]	n.a.[3]
	WBC count (cells/mm^3)	877	8,880 (10,600, 6,400)	386	8,800 (7,300, 10,700)	895	8,880 (7,490, 10,600)	n.a.[3]	n.a.[3]
	Absolute CD4 T-cell (cells/mm^3)	814	410 (280, 550)	358	378.5 (250, 540)	810	410 (280, 550)	4	565 (417, 853)
	Absolute CD4 T-cell among pregnant women (cells/mm^3)	810	409.5 (280, 550)	358	378.5 (250, 540)	810	410 (280, 550)	-[2]	-[2]

Note: [1] 46 of 399 subjects participating in the study on a prevention of perinatal HBV transmission were subtracted; [2]Not applicable; [3]Not available.
